# Uterine Sandwich Method: A Case of Posterior Placenta Previa in an In Vitro Fertilization Pregnancy Complicated by Velamentous Cord Insertion

**DOI:** 10.7759/cureus.8525

**Published:** 2020-06-09

**Authors:** Joseph Farshchian, Martin Castaneda

**Affiliations:** 1 Surgery, Florida Atlantic University College of Medicine, Boca Raton, USA; 2 Obstetrics and Gynaecology, Bethesda Hospital East, Boynton Beach, USA

**Keywords:** obstetrics, gynaecology, postpartum hemorrhage, uterine sandwich, b-lynch suture

## Abstract

The risk of postpartum hemorrhage (PPH) and placental adhesion anomalies, including placenta previa, may be increased in pregnancies conceived by in vitro fertilization (IVF) and other forms of assisted reproduction technologies. The uterine compression suture, known as the “uterine sandwich method,” may be useful in pregnancies complicated by placenta previa. We report an unusual case of placenta previa complicated by velamentous cord insertion, which was treated by a B-Lynch suture, a Bakri balloon tamponade, and vaginal packing.

## Introduction

Placenta previa is a complication of placental adhesion to the uterine wall, where placental tissue extends over the internal cervical os. This pathology increases the risk of preterm delivery, as well as fetal and maternal morbidity [1]. Placenta previa places mothers at risk for postpartum hemorrhage, a significant factor in maternal mortality and morbidity. The common placental anomalies include placental abruption, placenta previa, adherent (accreta, increta, percreta), and retained placenta. Velamentous cord insertion (VCI) is an abnormal cord insertion in which umbilical vessels diverge as they traverse between the amnion and chorion before reaching the placenta [2]. These vessels lack protection from Wharton’s jelly and are, therefore, more prone to compression and rupture. VCI pregnancies are at greater risk for placenta previa as well as other adverse perinatal outcomes. A three-fold higher risk of placenta previa has been seen in pregnancies conceived by in vitro fertilization (IVF) [3].

The B-Lynch suture is used to prevent postpartum hemorrhage (PPH) in uterine atony. It has also been used in cases of placental abnormalities [4]. No prior reports have described the use of B-Lynch sutures and the Bakri balloon in a case of posterior placenta previa with velamentous cord insertion. We aim to describe the techniques used in this case to supplement established management protocols of pregnancies complicated by placenta previa and abnormal cord insertions.

## Case presentation

A 35-year old woman, gravida 4, abortus 2, at 37 weeks gestation presented for her scheduled c-section. A week prior, an ultrasound showed no changes in the patient’s previously diagnosed posterior placenta previa. The patient had been followed by maternal-fetal medicine due to her high-risk pregnancy (IVF conception, placenta previa, and velamentous cord insertion), which lead to the decision of performing a cesarean section at 37 weeks gestation. Past medical history included chronic pelvic inflammatory disease and hypothyroidism, the latter controlled by 50 mcg levothyroxine daily. Non-stress test evaluation demonstrated a fetal heart rate of 140 beats per minute. Her preoperative vital signs were: Blood pressure 120/60 mmHg, heart rate 90 beats per minute, temperature 36.8°C, and respiratory rate 18 respirations per minute. Laboratory testing was unremarkable. The patient was taken to the operating room and spinal anesthesia was given. A lower segment cesarean section was performed and a 3170-gram baby with Apgar scores of 9 and 9 at one and five minutes, respectively, was delivered. The placenta was found to extend over the internal cervical os and posterior uterus. After a 30-second delayed cord clamping, cord blood was collected and Pitocin started. The patient also received a dose of methergine and tranexamic acid for bleeding control. The placenta slowly delivered spontaneously. The uterus was exteriorized and cleared of clots of debris. At this point, there was blood oozing noted from the lower uterine segment. A Bakri balloon was then utilized. The balloon was placed through the uterine incision while the catheter portion was guided through the cervix into the vaginal canal. The uterine incision was then closed with a #1 chromic in running locked fashion and the second layer using the same stitch. The second layer was closed with interrupted mattress sutures along the incision to obtain good hemostasis. The uterine tone was found adequate at this point, however, the decision was made to fill the Bakri balloon with 240 mL normal saline and to tie down the B-Lynch suture. The posterior cul-de-sac was cleared of all clots and debris, and the ovary and fallopian tubes were inspected and appeared normal. The patient tolerated the procedure well and was taken to the recovery room in satisfactory condition. The Bakri balloon and vaginal packing were taken out the morning after the surgery. The patient’s hemoglobin was 10.8 g/dl on post-op Day 1 with no evidence of postpartum bleeding or hemorrhage. The patient was discharged three days later without any complications.

## Discussion

PPH is a serious obstetric emergency that is a leading factor of maternal morbidity and mortality. The hemorrhage may occur after vaginal delivery or cesarean section. The etiologies of PPH include uterine atony, placental adhesion, placental retention, genital tract lacerations, and coagulopathies. PPH has been reported in as many as 4%-6% of pregnancies [1]. Conservative management includes uterine massage and compression, uterotonics, and balloon tamponade, however, PPH may progress to require surgical interventions such as uterine artery embolization or hysterectomy. Many obstetricians choose a combination of therapies to ensure the prevention and control of PPH.

Placenta previa, a disorder of placenta adhesion, occurs when the placenta covers the internal cervical os. The prevalence rate has been described as 4.1 per 1000 births [5]. It is another main cause of maternal morbidity and mortality. Risk factors include advanced maternal age, grand multiparity, history of previous cesarean section, IVF pregnancy, previous abortion, and smoking during pregnancy [6]. The presence of velamentous cord insertion also placed this patient at a higher risk of obstetric complications. Abnormal cord insertions are associated with cesarean deliveries and warrant raising suspicion of a high-risk pregnancy and possible vasa previa [2].

**Figure 1:**
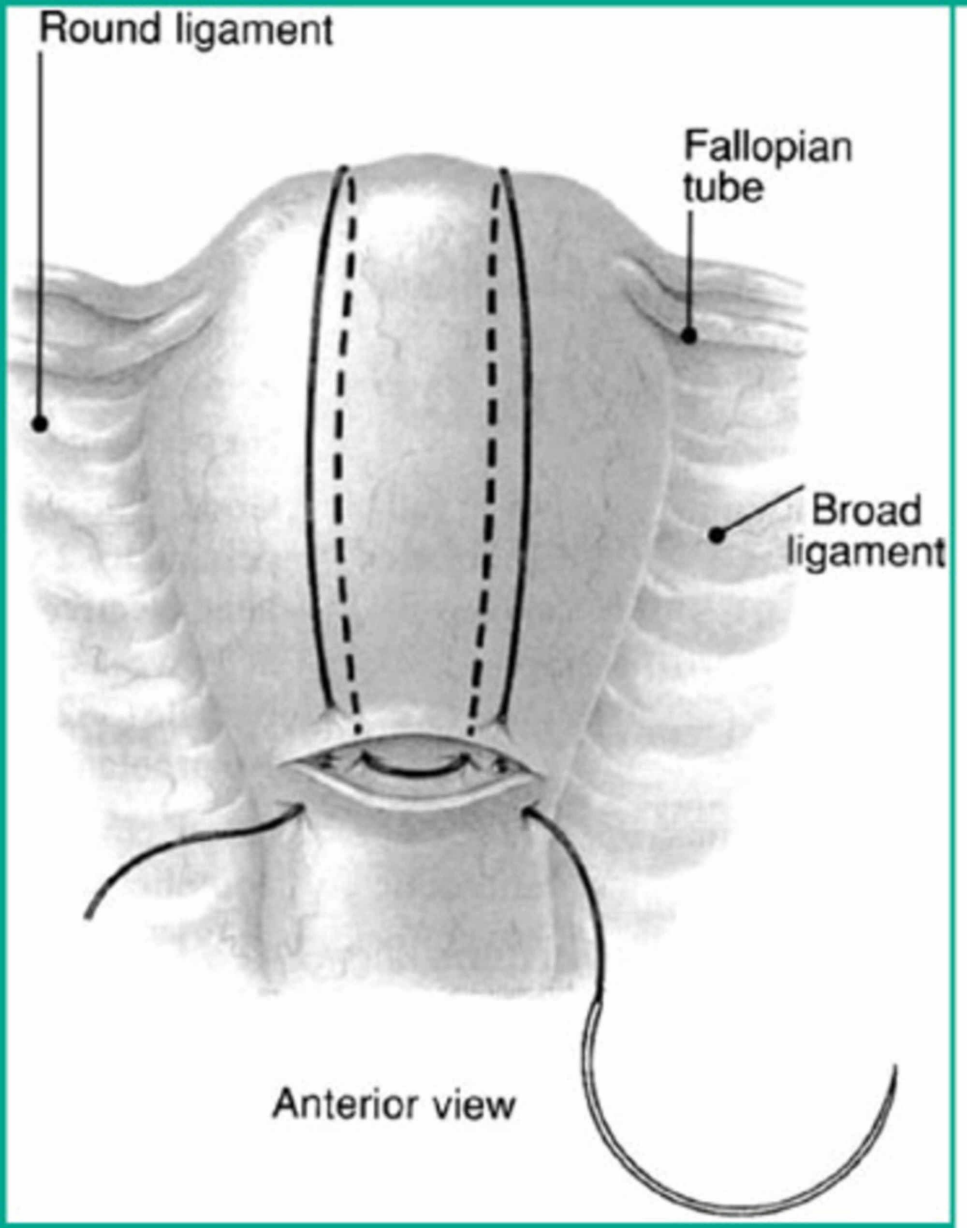
The B‐Lynch suture, from Lynch et al.

The B-Lynch suture (Figure 1) has been found to be highly successful in arresting cases of PPH resulting from uterine atony and an alternative to hysterectomy [7]. Particularly, it is a useful suture in controlling bleeding from placenta previa or placenta accreta. In addition, the use of a tamponade balloon, such as the Bakri, has been shown to be effective in controlling PPH originating from the placental site [8]. The balloon can be secured by packing the vaginal vault with sterile gauze or lap sponge after insufflation. Particularly, the balloon is effective for lower segment bleeding [9].

## Conclusions

Placenta previa and velamentous cord insertion increase the risk of PPH, particularly in patients with multiple risk factors such as in the reported patient. In high-risk pregnancies with multiple complications, it is important for surgeons to be aware of their therapeutic options. Here, a combination of the discussed procedures was enlisted to ensure minimal risk of complications. In this rare case, the combination of B-Lynch sutures, Bakri balloon tamponade, uterotonics, and vaginal packing was sufficient in achieving hemostasis and preventing postoperative complications.
